# Fine Particle Sensor Based on Multi-Angle Light Scattering and Data Fusion

**DOI:** 10.3390/s17051033

**Published:** 2017-05-04

**Authors:** Wenjia Shao, Hongjian Zhang, Hongliang Zhou

**Affiliations:** State Key Laboratory of Industrial Control Technology, College of Control Science and Engineering, Zhejiang University, Hangzhou 310027, China; wjshao@zju.edu.cn (W.S.); hjzhang@iipc.zju.edu.cn (H.Z.)

**Keywords:** PM2.5, mass concentration, light flux, weather index, relative humidity

## Abstract

Meteorological parameters such as relative humidity have a significant impact on the precision of PM2.5 measurement instruments based on light scattering. Instead of adding meteorological sensors or dehumidification devices used widely in commercial PM2.5 measurement instruments, a novel particle sensor based on multi-angle light scattering and data fusion is proposed to eliminate the effect of meteorological factors. Three photodiodes are employed to collect the scattered light flux at three distinct angles. Weather index is defined as the ratio of scattered light fluxes collected at the 40° and 55° angles, which can be used to distinguish the mass median diameter variation caused by different meteorological parameters. Simulations based on Lorenz-Mie theory and field experiments establish the feasibility of this scheme. Experimental results indicate that mass median diameter has less effect on the photodiode at the 55° angle in comparison with photodiodes at the 40° angle and 140° angle. After correction using the weather index, the photodiode at the 40° angle yielded the best results followed by photodiodes at the 55° angle and the 140° angle.

## 1. Introduction

Fine particles with aerodynamic diameters of 2.5 μm or less (PM2.5) have received considerable attention because of their adverse impacts on human health, especially their ability to penetrate deep into our lungs and even blood streams. In 2013, the International Agency for Research on Cancer (IARC) classified Particulate Matter (PM) from outdoor air pollution as carcinogenic to humans [1]. PM2.5 has been listed in the revised Chinese National Ambient Air Quality Standards (NAAQS). The limit of daily average PM2.5 mass concentration is 35 μg/m^3^ while it is 10 μg/m^3^ inthe World Health Organization (WHO) air quality guideline. Monitoring networks have been established to record the levels of fine particulate matter (PM2.5) in China. There are several technologies that can be used to determine the PM2.5 mass concentrations, among which the Tapered Element Oscillating Microbalance (TEOM) and beta attenuation method are most popular. However, instruments based on these methods are quite expensive (>$20,000) and require regular maintenance and calibration. Many new particle sensors have emerged in recent years, which are based on particle charging [2], silicon carbide-field effect transistor(SiC-FET) technology [3], conductivity [4,5] and other techniques. Instruments based on light scattering, such as the DustTrak II Aerosol Monitor 8530, SidePak Aerosol Monitor AM510, and DC1700, can provide continuous PM2.5 mass concentrations at relatively low cost [6]. However, light scattering is affected by various factors, for example, the particle’s size, refractive index, density and shape. These instruments need proper calibration to achieve accurate measurement. Furthermore, these instruments do not perform well when the compositions of PM2.5 vary greatly. Therefore, most of these instruments are used indoors or in situations where particle composition remains constant. Numerous studies have been carried out to analyze the calibration of different kinds of PM2.5 [7,8,9]. It is a great challenge to accurately estimate PM2.5 mass concentration for diverse kinds of PM2.5.

Research has revealed that high Relative Humidity (RH) will have a strong effect on light scattering [10,11,12,13,14,15]. Ammonium sulfate and ammonium nitrate are the major semi-volatile components of PM2.5.The deliquescence point of ammonium sulfate and ammonium nitrate is 62% and 80%, respectively. Therefore, when RH is higher than 62%, particle-bound water increases. The particle’s mass will increase when RH exceeds 65% and will increase sharply when RH exceeds 80%, which will lead to the overestimation of PM2.5 [13]. Some researchers introduced the hygroscopic growth factor, which is the ratio of a particle’s diameter under high RH conditions to that under dry conditions, to assess the effect of RH on measurement results. The hygroscopic growth factor of particles in the range of 200 nm to 1 µm is about 1.5 when RH is 90% and reaches nearly 2.0 when RH is 95%. A little variation in RH can result in considerable changes in the hygroscopic growth factor, especially when RH exceeds 90% [16]. High RH will enlarge particle size and reduce the particle refractive index, resulting in a more smooth or spherical shape through the absorption of water by particles. Countless efforts have been made to eliminate the effects of RH. Sioutas et al. used a diffusion dryer to exclude aqueous components of PM2.5 in real-time PM2.5 mass concentration measurements [11]. However, adsorbed water needs to be driven off the desiccant in the diffusion dryer every 7 h. Others used heated inlets to remove aqueous components, which will cause excessive volatilization of labile PM2.5 constituents. Thus, additional instruments should be added to determine the mass of volatile component in PM2.5. For example, a Filter Dynamic Measurement System (FDMS) should be incorporated into a conventional TEOM monitor, which evaporates condensed water, to ensure the reliability of PM2.5 measurement results [17].

In order to diminish the influence of meteorological parameters such as RH on the PM2.5 sensors based on light scattering, a new particle sensor based on multi-angle light scattering and data fusion is proposed in this paper. The sensor presented here is a new version of the prototype developed by our group and shows better accuracy and stability with respect to thedevice reported earlier in reference [18]. The rest of the paper is arranged as follows: the design of the sensor is described in Section 2. Theory of data fusion and the signal processing method is described in Section 3. In Section 4, field experiments are carried out to verify the validity as well as evaluate the performance of the particle sensor. The final section, Section 5, summarizes the characteristics of the particle sensor.

## 2. Design of the Sensor and Particle Measurement System

In order to overcome the drawbacks of the existing PM2.5 sensors based on light scattering, a new particle sensor with more than one optical detector was developed. Simulations were performed to obtain the appropriate parameters of the sensor. The distribution of the scattered light of a particle varies with the particle’s diameter, refractive index and shape. The Lorenz-Mie solution of Maxwell’s equations describes the scattering of an electromagnetic plane wave by a homogeneous sphere [19]. To optimize the mounting angle of the optical detector, scattered light flux collected by the optical detector is calculated for particles with different refractive indices and diameters [20]. The following simulation conclusions can be drawn: (1) forward scattered light flux is insensitive to the imaginary part of particle’s refractive index, which is consistent with conclusions in [21], (2) sideward scattered light flux is insensitive to the real part of a particle’s refractive index, and (3) backward scattered light flux is sensitive to a particle’s shape.

**Figure 1:**
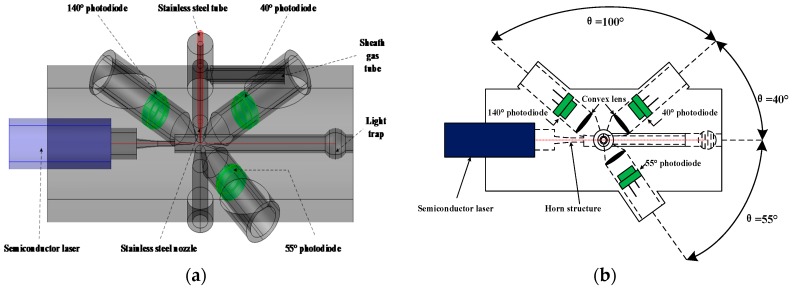
Diagram of the particle sensor: (**a**) 3D view; (**b**) Top-down view.

A 3D view of the particle sensor is shown in Figure 1. The main body of the sensor, with the size of 120 mm × 80 mm × 80 mm, is produced by a 3D printer using photosensitive resin. Both the inside and outside walls of the main body are painted with black matt varnish to minimize the reflection of the scattered light and the effect of stray light. A light trap is constructed on the right side to suppress stray light from the light source. A stainless steel tube and a nozzle are introduced to reduce the settlement of particles in the gas tube and to focus the particle flow into the laser beam. The nozzle is also coated with black matt varnish to reduce stray light. A semiconductor laser is used to emit a 648 nm light beam in continuous wave mode. Air flow containing particles enters the optical chamber via the stainless steel tube, where it is illuminated by the laser beam. Scattered light in the scattering angle ranges of 40° ± 30°, 55° ± 30° and 140° ± 30° is captured by three convex lenses and then focused onto three photo-detectors. Akey element of the photo-detectors is photodiode S2386-44K (Si-PD, Hamamatsu Photonics, Hamamatsu, Japan) [22]. A horn structure is used to prevent the stray light of the laser beam from entering into the particle sensor and to stably maintain the power of the laser beam stable. A sheath gas tube is reserved to protect the optical systems from particle pollution. The diameter of the tube is optimized to ensure small particles can be detected and stray light owing to reflections off surfaces of the tube can be suppressed.

**Figure 2:**
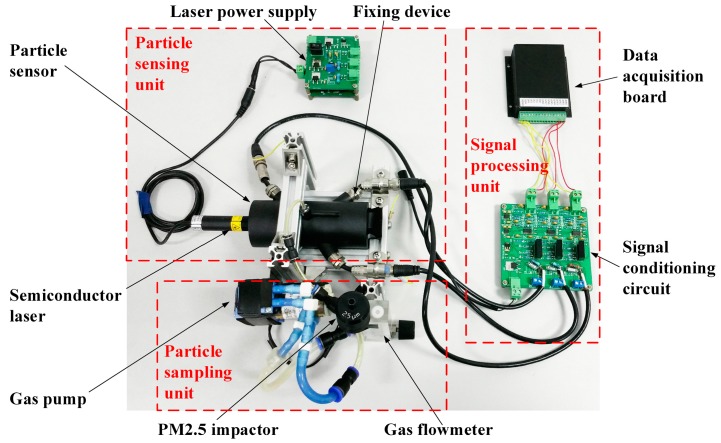
Composition of the particle measurement system.

**Figure 3:**
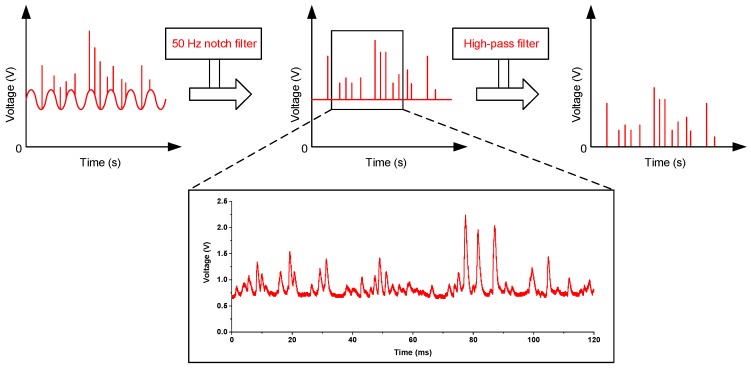
Signal processing steps.

The particle measurement system consists of the particle sampling unit, particle sensing unit and signal processing unit, as shown in Figure 2. The particle sampling unit comprises a PM2.5 impactor (TSI Inc., Shoreview, MN, USA), a miniature diaphragm gas pump D871-23 (Parker Inc., Cleveland, OH, USA) and a gas flow meter (Senlod Inc., Nanjing, China). The PM2.5 impactor is used to pre-condition the size range of the particles entering the particle sensor. By controlling the pump speed at 3.0 L/min, the impactor can isolate particles with aerodynamic equivalent diameter less than 2.5 μm from coarse particles in the particle sensing unit. A multi-coil transformer and a LDO voltage regulator TPS 78633 are used to build up high-quality laser power supply. The signal processing unit is mainly a current-to-voltage converter. Weak current from the photodiodes is amplified by LMP 7721, an operational amplifier with ultra-low noise and high precision. The stray light acts as a kind of continuum, which changes slowly over time due to changes in temperature and pressure conditions or possible dust contamination in the optical chamber. The light scattered by the particles is superimposed on this continuum. The stray light signal can be assumed as a continuous base-line over a short time interval. As shown in Figure 3, a 50 Hz notch filter is employed to remove power supply noise, and a high-pass filter is used to remove stray light voltage signal in the signal conditioning circuit. Then, the scattered light signal sare acquired by an eight-path synchronous data acquisition card with a sample rate of 12.5 KHz and processed using LabVIEW.

**Figure 4:**
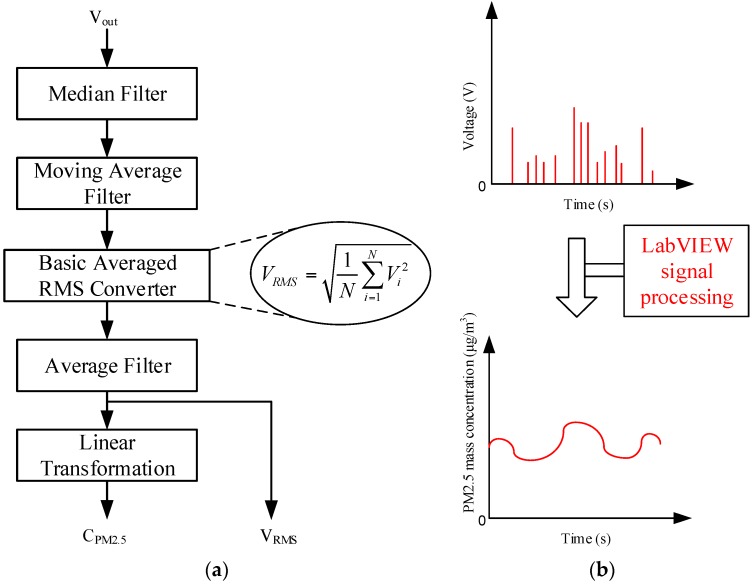
Schematic diagram of software signal processing: (**a**) flow diagram; (**b**) waveforms.

Similar to the RMS (Root Meam Square) moving average method in reference [18], a signal processing program was written using LabVIEW. As illustrated in Figure 4, the scattered light signals of the three photodiodes are conditioned by Median Filter, Moving Average Filter and so on. The rectangular window is applied to the record scattered light signals before RMS computation. In addition, the averaging time of the RMS value is one second. Mass concentrations as well as voltage signals are recorded once per second.

## 3. Theory of Data Fusion

An empirical Correction Factor (CF) was used to compensate the influence of RH [10,12,23]. However, empirical CF values vary greatly with particle composition and size distribution. Moreover, an empirical formula will introduce substantial deviation when RH cannot be measured precisely. Other than measuring RH or adding extra equipment to remove particle-bound water, the discrepancy of the scattering pattern under different RH conditions can be used to correct PM2.5 measurements. According to Lorenz-Mie theory, when a particle’s diameter increases, the scattering light will turn to the forward direction. Multi-angle light scattering can be used to account for the effect of the hygroscopic growth factor and other weather conditions. A multi-angle light scattering technique has been used in the particle measurement field and can be used to distinguish the type of weather [24,25] as well as the typology of aerosols [26,27].

Based on the above analysis, the Weather Index (WI), defined as the ratio of scattered light flux collected by photodiodes at two forward angles, is proposed to distinguish different weather conditions, especially different RHs, as shown in Equation (1).

(1) $$WI=\frac{F(40{}^{\circ})}{F(55{}^{\circ})}$$

where F(40°) and F(55°) are scattered light flux collected by the photodiodes at 40° and 55° angles, respectively, in the particle sensor.

High RH will lead to a considerable hygroscopic growth factor and then a larger diameter for a single particle and a larger MMD (Mass Median Diameter) for a particle group. Simulations based on Lorenz-Mie theory are used to find the relationship between particle size distribution and WI.

The angular scattered light flux of a single particle is calculated as follows:

(2) $$F(\theta ,D,m)=\int_{\varphi_{1}}^{\varphi_{2}}d\varphi \int_{\theta_{1}}^{\theta_{2}}I_{s}(D,m,\lambda )r^{2}\sin\theta d\theta$$

where φ is the azimuth angle between the scattering plane and polarization direction, θ is the scattering angle, $I_{s}$ is scattering light intensity at one point in space, λ is the wavelength of the light source, D is the particle diameter, m=n+ki is a complex refractive index of the particle, n and k are the real and imaginary parts of the refractive index respectively.

The log-normal distribution is often used to estimate the particle size distribution of aerosols. A particle’s diameter D is log-normally distributed if the logarithm of D and the particle’s volume V are normally distributed,

(3) $$\frac{dV}{V}=\frac{1}{\sqrt{2\pi }\ln\sigma_{g}}\exp\left[\frac{-{\left(\ln D-\ln VMD\right)}^{2}}{2{\left(\ln\sigma_{g}\right)}^{2}}\right]d\ln D$$

VMD and $\sigma_{g}$ are volume median diameter and geometric standard deviation, respectively.

For a group of particles, angular scattering flux is calculated as follows:

(4) $$F(\theta ,m)=\sum_{i=1}^{i=N}P(D_{i})F(\theta ,D_{i},m)$$

$P(D_{i})$ is the ratio of the volume of a particle with diameter $D_{i}$ to the total volume of particles in the group:

(5) $$P(D_{i})=\frac{V(D_{i})}{\sum_{i=1}^{i=N}V(D_{i})}$$

The typical size distribution of fine particle matter (PM2.5) is log-normal, the MMD is 0.3 μm, the geometric standard deviation is 2.0. VMD (Volume Median Diameter) and the MMD is the same if all the particles are assumed to have the same mass density. MMD of aerosol sulfate is typically 0.54 μm and 0.20 μm [28]. The aerosol size distribution of Los Angeles Smog is bimodal, and the size distribution in PM2.5 size range is a log normal distribution with an MMD of 0.3 μm and a geometric standard deviation of 2.25 [29]. In the simulations, MMD will vary from 0.05 μm to 2.5 μm while the geometric standard deviation will remain at 2.0. Single particles and particle groups are both considered. The real part of the refractive index will vary from 1.41 to 1.51, and the imaginary part of the refractive index will vary from 0.00 to 0.15. Considering carbonaceous particles, particles with a high refractive index are also taken into consideration.

**Figure 5:**
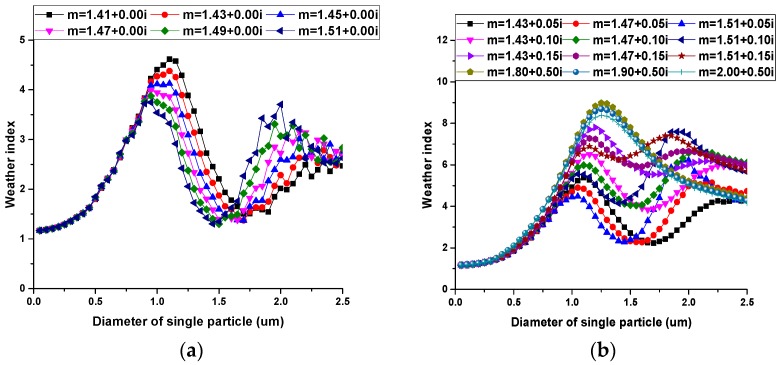
Relationship between the diameter of single particle and the weather index: (**a**) non-absorbing particles; (**b**) absorbing particles.

**Figure 6:**
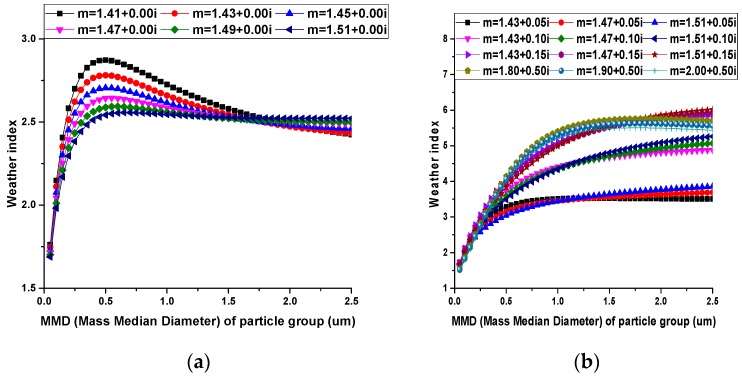
Relationship between MMD (Mass Median Diameter) of particle groups and the weather index: (**a**) non-absorbing particles; (**b**) absorbing particles.

Simulation results are shown in Figure 5 and Figure 6.

From Figure 5, it can be concluded: (1) For a single particle, the weather index increases with diameter and reaches the maximum value at about 1.0 μm; (2) the weather index of absorbing particles is larger than that of non-absorbing particles; and (3) the weather index decreases with the real part of the refractive index when theparticle’s diameter is between 1.0 μm and 1.5 μm.

From Figure 6, similar conclusions can be drawn: (1) For a group of non-absorbing particles, the weather index increases sharply with MMD and reaches the maximum value when MMD is about 0.6 μm and then decreases slowly. (2) For a group of absorbing particles, the weather index increases with MMD and the weather index decreases with the real part of the refractive index and increases with the imaginary part of the refractive index when MMD is between 0.5 μm and 1.0 μm.

Water absorbs slightly in the visible spectrum, and the absorption will become stronger with wavelength [30]. The operating wavelength of the laser is 648 nm, and the refractive index of water is m = 1.33 + 1.5 × 10^−8^i, so laser light absorbed by water can be neglected.

As mentioned above, high RH will have three effects on the particle’s properties: (1) particle size increases with the hygroscopic growth factor, (2) a particle’s real part of the refractive index decreases by adding the lower refractive index of the water, (3) a particle’s shape tends to becomesmoother by absorbing water. According to the simulation results above, as a whole, high RH will result in a larger WI by making MMD larger, and the real part of the refractive index of the particle group will become smaller. Furthermore, the effect of MMD is much more significant than that of the refractive index. Our simulation results are in agreement with, e.g., Sioutas et al., who reported in reference [11] that the aerosol MMD is the single most important parameter in affecting the response of PM2.5 measurement instruments based on light scattering, and the effect of particle chemical composition on PM2.5 measurement results is much less important than particle size distribution. Therefore, the weather index can be used to correct the PM2.5 measurements.

## 4. Results and Discussion

**Figure 7:**
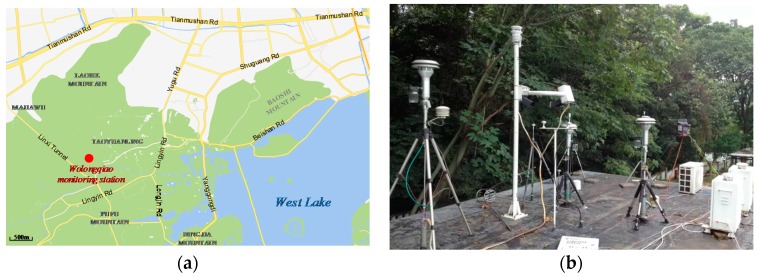
Wolongqiao monitoring station: (**a**) location; (**b**) appearance.

Field experiments were conducted at the Wolongqiao monitoring station, anational monitoring station of the National Ambient Air Quality Automatic Monitoring Network, operated by the Ministry of Environmental Protection of China. The station is situated at 30°14′44″ N, 120°07′37″ E. As illustrated in Figure 7, the station is surrounded by forests and is suitable for studies on the meteorological effect of PM2.5 monitoring. There is a city expressway to the north side of the station and a tunnel to the southwest of the station. Automobile exhaust is one of the main sources of air pollution on weekends and holidays, while during the working days, air quality near the station is better than other areas of the city. The station is equipped with a TEOM 1405-D monitor (Thermo Fisher Scientific Inc., Waltham, MA, USA, with a precision of ±2.0 μg/m^3^) to measure PM2.5 and PM10 mass concentrations simultaneously and can be used as a reference PM2.5 instrument in field experiments.

**Figure 8:**
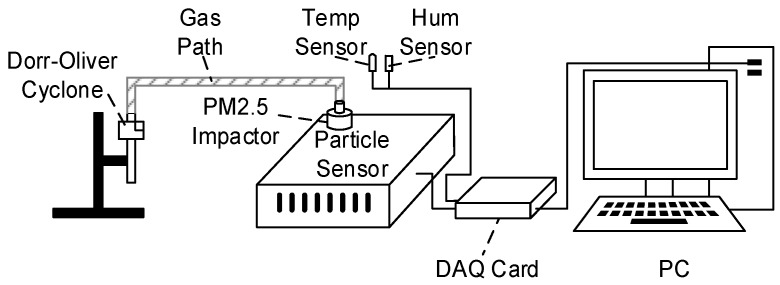
Schematic diagram of the experimental system.

The experimental system is shown in Figure 8.The air is introduced into a PM2.5 impactor through a gas path after filtering the coarse particles using a Dorr-Oliver cyclone. A commercial temperature sensor with a precision of ±0.5 °C and an RH sensor with a precision of ±3% RH are used to measure temperature and RH, respectively. The particle sensor is assembled into a metal chassis, which can effectively suppress electromagnetic interference and environmental noises. The output signals from the particle sensor were acquired using a DAQ (Data Acquisition) card (Daqvantech Inc., Suzhou, China) with a sample rate of 12.5 KHz. Then, the data were processed and displayed on a PC.

The new particle sensor was tested at the Wolongqiao monitoring station from 7 to 14 March 2016. Hourly PM2.5 and PM10 mass concentrations from the TEOM 1405-D monitor were recorded simultaneously. Half-hourly meteorological parameters such as visibility, barometric pressure, wind speed, wind direction and weather types from a nearby weather station were also recorded. Before experiments are conducted, zero-point calibration needs to be conducted using a HEPA (High Efficiency Particulate Air) filter (TSI Inc., Shoreview, MN, USA).

**Figure 9:**
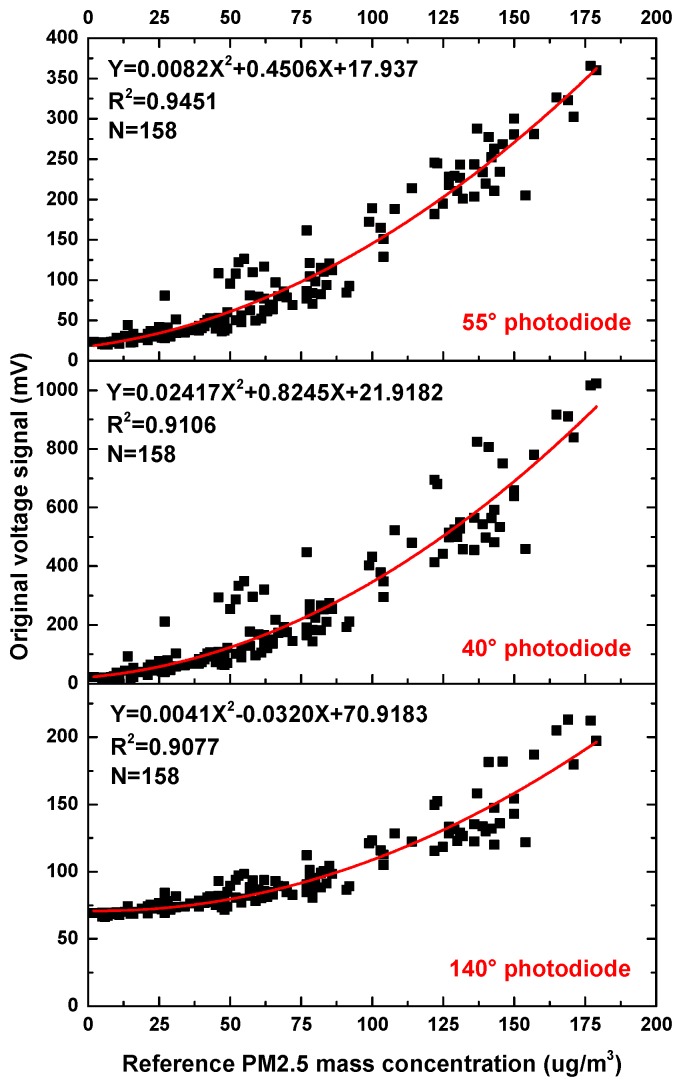
Output voltage of the three photodiodes in the particle sensor vs. 1-h average reference PM2.5 mass concentration.

Figure 9 shows the relationship between the output voltages of the three photodiodes in the particle sensor with 1-h average PM2.5 mass concentrations given by TEOM 1405-D. Quadratic polynomial regression is adopted considering the nonlinearity. The fitting degree of curvilinear regression is examined by coefficient of determination (R^2^) and root mean square error (RMSE). There is a significant correlation between the output of the photodiodes and the PM2.5 mass concentration.

**Figure 10:**
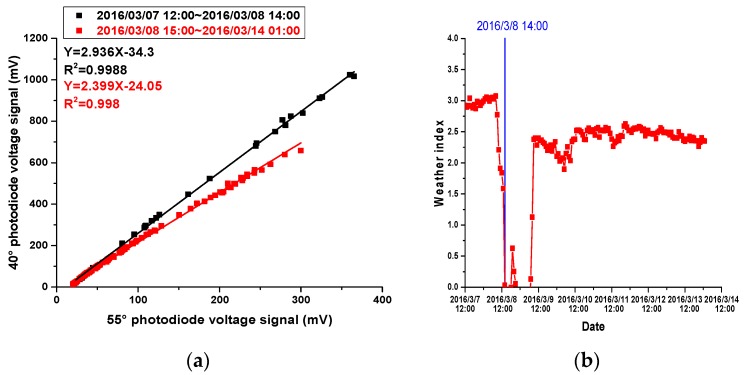
Parameters of the weather index: (**a**) Scatter plot of the 55° photodiode voltage signals vs. the 40° photodiode voltage signals, (**b**) Variation of the weather index during the measurement period.

As illustrated in Figure 10, the scatter plot of the 1-h average PM2.5 mass concentrations based on the 55° photodiode and the 40° photodiode in the particle sensor follow two distinct trends. According to the variation of the weather index, the weather index is relatively stable, i.e., approximately 3.0 for the first 20 h, and then drops sharply below 0.0 at 8 March 2016 14:00 with decreasing PM2.5 mass concentrations; then, the weather index increases sharply to about 2.5 and becomes stable until the end of the measurement. One of the possible reasons that the weather index can be less than zero is that the 40° photodiode does not receive a sufficient voltage signal when the PM2.5 mass concentration is very low (e.g., less than 20 μg/m^3^) and the voltage of the 40° photodiode can even be negative when system noise voltage is removed from photodiode voltage. This abnormal phenomenon only occurs when the PM2.5 mass concentration is less than 20 μg/m^3^ and should be considered separately. One of the possible solutions is to use the traditional PM2.5 mass concentration of the 40° photodiode (linear transformation of the 40° photodiode voltage signal) when the PM2.5 mass concentration is less than 20 μg/m^3^. Because the particle size distribution can only change slowly, data points are broken into two continuous parts according the value of the weather index. The two parts are separated by 8 March 2016 at 14:00 h. Both parts of the data points were analyzed by linear regression, and the results were excellent, indicating that during different periods of time, parameters of the PM2.5 particles are distinct, while parameters are stable during the same period of time.

**Figure 11:**
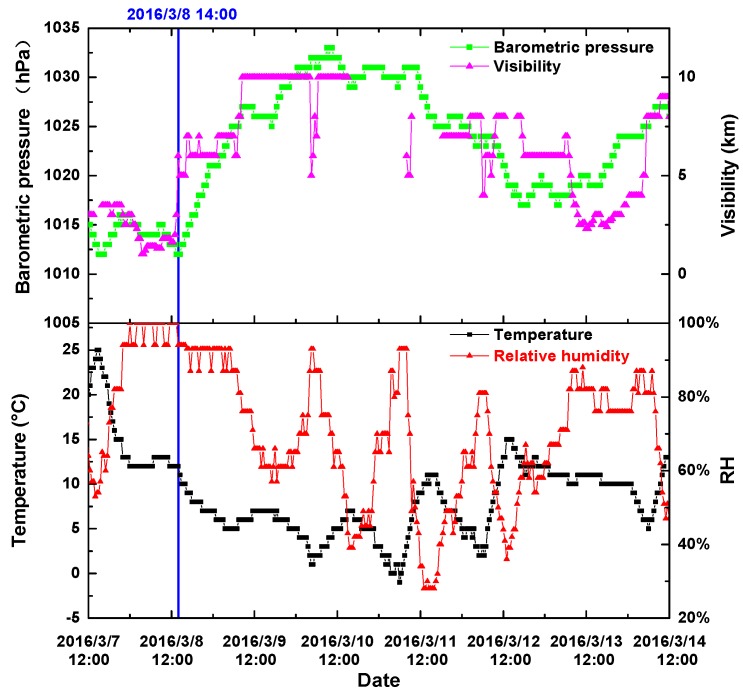
Meteorological parameters during the measurement period: Temperature, RH, visibility and barometric pressure.

Variations of the meteorological parameters during the experiment are illustrated in Figure 11. During the former period of time, temperature is higher than 12 °C; RH is mostly higher than 93% RH; visibility is less than 3 km; and barometric pressure is lower than 1015 hPa, while during the latter period of time, temperature changes significantly but remains below 12 °C; RH also varies greatly, but remains lower than 93% RH; visibility and barometric pressure follow almost the same trend; visibility is greater than 3 km; and barometric pressure is higher than 1015 hPa during most of the time. It is obvious that meteorological parameters during the former period of time are quite different from those during the latter period of time. Therefore, it is reasonable that the weather index can help to distinguish meteorological parameters. MMD is the most important factor that affects the measurement of instruments based on light scattering [11]. It is obvious that MMD during the two periods of time is quite different. From the simulation results presented in Chapter 3, a high WI indicates a large MMD. Therefore, during the former period of time, a larger MMD can be estimated, while during the latter period of time, a smaller MMD is reasonable. According to the experimental results of other researchers, when MMD varies between 0.3 μm and 1.0 μm, the ratio of particle measurement results based on light scattering method to results based on the dynamic weighing method will increase with increasing MMD [11].

**Figure 12:**
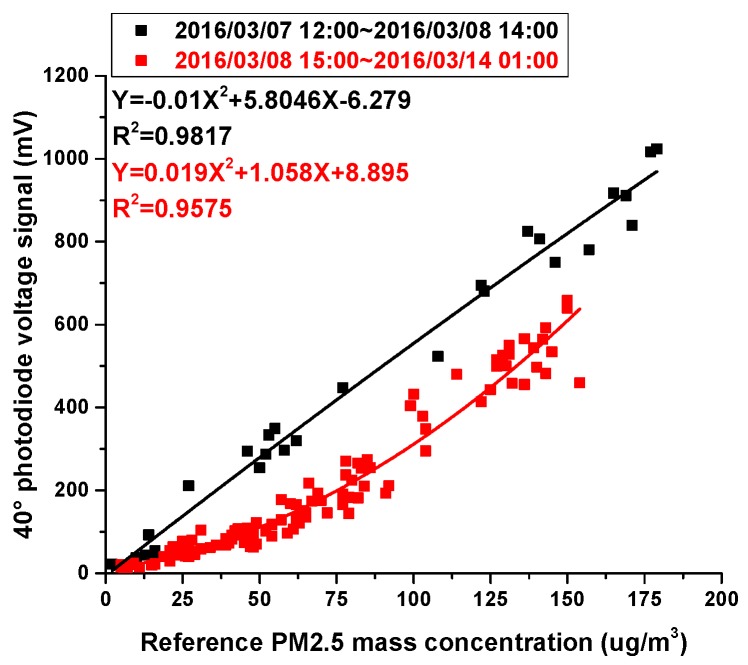
Relationship between PM2.5 mass concentrations measured using the 40° photodiode and reference PM2.5 mass concentrations at Wolongqiao monitoring station.

In Figure 12, the correction of PM2.5 mass concentrations is displayed using the 40° photodiode as an example. According to the weather index, the points of the scatter plot are divided into two groups: the black ones and red ones. For different groups, a unique quadratic polynomial regression model is used to describe the relationship between the output voltage of the photodiode at the 40° angle and the reference PM2.5 concentrations. The results are very good, with R^2^ ranging between 0.9817 and 0.9575. During the former period of time, the data points follow a linear trend while during the latter period of time, the data points follow a non-linear trend. It is not unusual. During the whole experiment, we used an index of 1.5 to describe the nonlinear relationship between scattered light flux and corresponding particle volume, as illustrated in the following expression.

(6) $$V\propto F^{1.5}$$

where *V* is the particle volume and *F* is scattered light flux.

This index is based on the theory of Fraunhofer diffraction, which is valid for particles larger than 2 μm and is widely used in PM10 measurement instruments [31,32]. Therefore, this index can be accurately applied when a larger MMD is estimated. However, the results will show nonlinearity when a smaller MMD is estimated.

The following example demonstrates how the WI is used to correct the 40° photodiode voltage signal.

When WI is higher than 2.7, the linear equation Y=0.1807X+0.9104 is used to correct the 40° photodiode voltage signal and obtain adjusted PM2.5 mass concentrations of the 40° photodiode for the former period of time; X is the 40° photodiode voltage signal, and Y is the adjusted PM2.5 mass concentration.

When WI is equal to or less than 2.7, the equation $Y=-0.0003148X^{2}+0.4109X+6.4123$ is used to correct the 40° photodiode voltage signal and obtain adjusted PM2.5 mass concentrations of the 40° photodiode for the latter period of time.

Adjusted PM2.5 mass concentrations for all experimental processes can be calculated based on these two equations.

**Figure 13:**
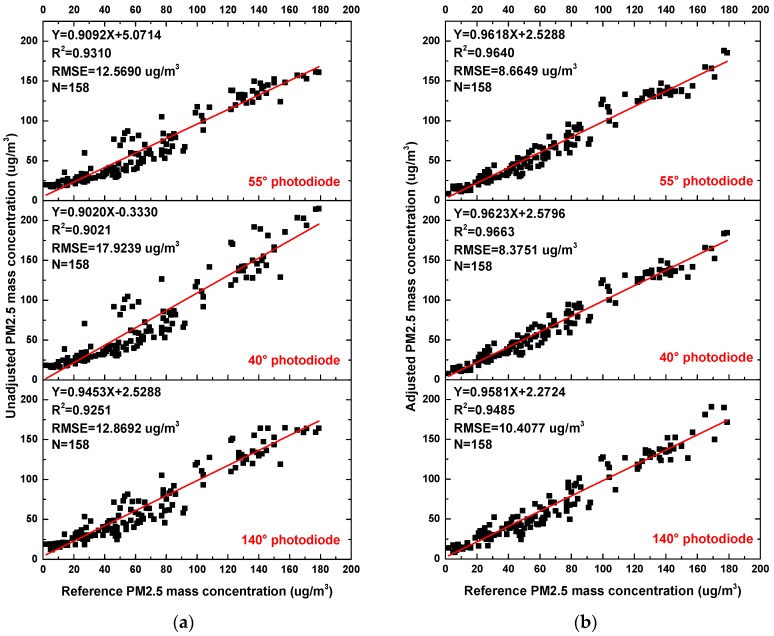
Scatter plot of the 1-h average PM2.5 mass concentrations using the reference Tapered Element Oscillating Microbalance (TEOM) 1405-D monitor at Wolongqiao monitoring station vs. PM2.5 mass concentrations based on three angular photodiodes in the particle sensor: (**a**) unadjusted; (**b**) adjusted.

Figure 13 displays the relationship between PM2.5 mass concentrations of three photodiodes and reference TEOM 1405-D monitored 1-h average PM2.5 mass concentrations before and after correction. It can be seen that the measurement accuracy and linearity are improved greatly after correction. Photodiodes at the angles of 40° and 55° perform very well. These results are consistent with results using an expensive optical particle counter (Grimm 1.108) (R^2^ = 0.97) [33] and better than field experiments using an aerosol monitor DustTrak (R^2^ = 0.859) [34]. In summary, PM2.5 mass concentrations measured by the three photodiodes are similar if MMD is stable. When MMD varies, the measurements will be affected. After correction by WI, photodiodes at the angle of 40° and 55° can result in a favorable performance.

As illustrated in Table 1, after correction by the WI, the three photodiodes can achieve accurate measurement of PM2.5 concentrations. However, there are still large biases in the three photodiodes, especially when PM2.5 concentrations are low. The minimum PM2.5 concentrations of the three photodiodes are about 8 μg/m^3^ while the minimum TEOM 1405-D is 2 μg/m^3^.The possible reason may be that the measurement model, which describes the relationship between the PM2.5 concentration and output voltage of the photodiode, does not fit the data well when the PM2.5 concentration is low.

**Figure 14:**
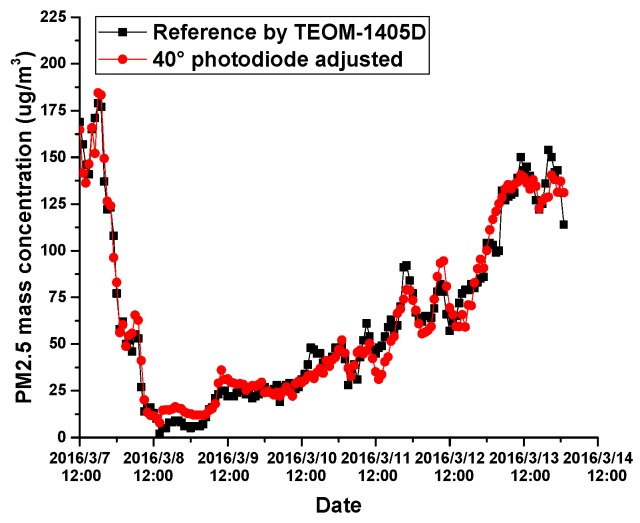
Comparison of 1-h average PM2.5 mass concentrations obtained simultaneously using a TEOM 1405-D monitor at Wolongqiao monitoring station and adjusted PM2.5 mass concentrations based on the 40° photodiode in the particle sensor according to different weather indices (the regression results are presented in Figure 13b, center plot).

Corrected PM2.5 mass concentrations measured by the 40° photodiode according to different WI and reference PM2.5 mass concentrations are presented in Figure 14. It can be seen that corrected PM2.5 mass concentrations follow the trend of reference PM2.5 mass concentrations very well. Figure 14 clearly reveals several temporal PM2.5 concentration patterns during the experiment. The PM2.5 concentrations dropped sharply with fresh, clean air brought by a cold wave from the northwest during the evening of 7 March 2016 until noon on 8 March 2016. Then, the PM2.5 concentrations increased slowly with temperature, and the effect of the cold wave vanished.

## 5. Conclusions

In this paper, a novel particle sensor based on multi-angle light scattering and data fusion is presented. In the design of the particle sensor, a weather index is introduced to describe MMD variation of PM2.5 particles caused by different meteorological parameters such as RH. High RH will enlarge the particle diameter significantly. Simulations are carried out to demonstrate that the WI can distinguish the variation of MMD caused by diverse meteorological parameters. The simulation results indicate that a larger MMD introduced by high RH will produce a higher WI while the particle refractive index has less impact on the WI.

Field experiments were carried out to further verify the ability of the weather index to distinguish the variation of MMD caused by different meteorological parameters. The field experiment results demonstrated that for a larger MMD, a linear relationship occurs between the values measured by the three photodiodes in the particle sensor and the reference TEOM results. However, for a smaller MMD, there is a nonlinear relationship between the values measured by the three photodiodes and the reference TEOM results. One possible reason is that the index used in the signal processing of the particle sensor is widely used in PM10 measurement instruments. Therefore, it is possible that the weather index can help to adjust the index to reduce the impact of MMD in further research.

Experimental results showed that the photodiode at the 55° angle can achieve good performance. After correction by the WI, photodiodes at 40° and 55° angles can both achieve good performance. This was presumably due to two reasons: (1) variations of the nonlinear relationship between scattered light flux and corresponding particle volume introduced by MMD variation are smallest for the 55° photodiode, while after correction by the weather index, the effect of MMD variation vanishes, and the effect of variation of a particle’s refractive index is smallest for the 40° photodiode; (2) the noise of the voltage signal is highest for the 140° photodiode and lowest for the 55° photodiode.

It should be noted that more work needs to be done to further improve the performance of the particle sensor. For example, more field experiments should be carried out to investigate the relationship between WI and MMD of particle groups. In addition, the measurement precision, as well as the detection limit, has yet to be improved. Furthermore, a virtual impactor is a potential alternative, which can relieve the maintenance work.

## Figures and Tables

**Table 1:** Summary of 1-h average PM2.5 measurements (μg/m^3^) by different instruments.

Instrument	Mean	Median	Minimum	Maximum
TEOM 1405-D	63.72	52.00	2.00	179.00
55° photodiode	63.81	45.86	8.57	188.13
40° photodiode	63.89	47.00	7.74	184.56
140° photodiode	63.32	47.20	8.20	190.68

## References

[1] Loomis D., Huang W., Chen G. (2014). The International Agency for Research on Cancer (IARC) evaluation of the carcinogenicity of outdoor air pollution: Focus on China. Chin. J. Cancer.

[2] Zhang C., Zhu R., Yang W.M. (2016). A Micro Aerosol Sensor for the Measurement of Airborne Ultrafine Particles. Sensors.

[3] Lloyd Spetz A., Huotari J., Bur C., Bjorklund R., Lappalainen J., Jantunen H., Schütze A., Andersson M. (2013). Chemical sensor systems for emission control from combustions. Sens. Actuators B Chem..

[4] Hagen G., Feistkorn C., Wiegartner S., Heinrich A., Bruggemann D., Moos R. (2010). Conductometric soot sensor for automotive exhausts: Initial studies. Sensors.

[5] Carminati M., Pedalà L., Bianchi E., Nason F., Dubini G., Cortelezzi L., Ferrari G., Sampietro M. (2014). Capacitive detection of micrometric airborne particulate matter for solid-state personal air quality monitors. Sens. Actuators A Phys..

[6] Sousan S., Koehler K., Thomas G., Park J.H., Hillman M., Halterman A., Peters T.M. (2016). Inter-comparison of low-cost sensors for measuring the mass concentration of occupational aerosols. Aerosol Sci. Technol..

[7] Jiang R.T., Acevedo-Bolton V., Cheng K.C., Klepeis N.E., Ott W.R., Hildemann L.M. (2011). Determination of response of real-time SidePak AM510 monitor to secondhand smoke, other common indoor aerosols, and outdoor aerosol. J. Environ. Monit..

[8] Dacunto P.J., Cheng K.C., Acevedo-Bolton V., Jiang R.T., Klepeis N.E., Repace J.L., Ott W.R., Hildemann L.M. (2013). Real-time particle monitor calibration factors and PM2.5 emission factors for multiple indoor sources. Environ. Sci. Process. Impacts.

[9] Dacunto P.J., Klepeis N.E., Cheng K.C., Acevedo-Bolton V., Jiang R.T., Repace J.L., Ott W.R., Hildemann L.M. (2015). Determining PM2.5 calibration curves for a low-cost particle monitor: Common indoor residential aerosols. Environ. Sci. Process. Impacts.

[10] Donateo A., Contini D., Belosi F. (2006). Real time measurements of PM2.5 concentrations and vertical turbulent fluxes using an optical detector. Atmos. Environ..

[11] Sioutas C., Kim S., Chang M.C., Terrell L.L., Gong H. (2000). Field evaluation of a modified DataRAM MIE scattering monitor for real-time PM2.5 mass concentration measurements. Atmos. Environ..

[12] Chakrabarti B., Fine P.M., Delfino R., Sioutas C. (2004). Performance evaluation of the active-flow personal DataRAM PM2.5 mass monitor (Thermo Anderson pDR-1200) designed for continuous personal exposure measurements. Atmos. Environ..

[13] Cropper P.M., Hansen J.C., Eatough D.J. (2013). Measurement of light scattering in an urban area with a nephelometer and PM2.5 FDMS TEOM monitor: Accounting for the effect of water. J. Air Waste Manag. Assoc..

[14] Baynard T., Garland R.M., Ravishankara A.R., Tolbert M.A., Lovejoy E.R. (2006). Key factors influencing the relative humidity dependence of aerosol light scattering. Geophys. Res. Lett..

[15] Karagulian F., Belis C.A., Lagler F., Barbiere M., Gerboles M. (2012). Evaluation of a portable nephelometer against the Tapered Element Oscillating Microbalance method for monitoring PM2.5. J. Environ. Monit..

[16] Liu P.F., Zhao C.S., Goebel T., Hallbauer E., Nowak A., Ran L., Xu W.Y., Deng Z.Z., Ma N., Mildenberger K. (2011). Hygroscopic properties of aerosol particles at high relative humidity and their diurnal variations in the North China Plain. Atmos. Chem. Phys..

[17] Grover B.D., Kleinman M., Eatough N.L., Eatough D.J., Hopke P.K., Long R.W., Wilson W.E., Meyer M.B., Ambs J.L. (2005). Measurement of total PM2.5 mass (nonvolatile plus semivolatile) with the Filter Dynamic Measurement System tapered element oscillating microbalance monitor. J. Geophys. Res..

[18] Shao W., Zhang H., Zhou H. A new particle sensor based on true RMS value measurement. Proceedings of the IEEEInternational Instrumentation and Measurement Technology Conference (I2MTC).

[19] Mie G. (1908). Pioneering mathematical description of scattering by spheres. Ann. Phys..

[20] Mätzler C. (2002). MATLAB functions for Mie scattering and absorption, version 2. IAP Res. Rep..

[21] Renard J.B., Thaury C., Mineau J.-L., Gaubicher B. (2010). Small-Angle Light Scattering by Airborne Particulates: Environnement S.A. Continuous Particulate Monitor. Meas. Sci. Technol..

[22] Datesheet of S2386-44K. http://www.hamamatsu.com/jp/en/product/category/3100/4001/4103/S2386-44K/index.html.

[23] Laulainen N. (1993). Summary of Conclusions and Recommendations from a Visibility Science Workshop.

[24] Li C., Peng P. (2012). Visibility measurement using multi-angle forward scattering by liquid droplets. Meas. Sci. Technol..

[25] Peng P., Li C.W. (2016). Visibility measurements using two-angle forward scattering by liquid droplets. Appl. Opt..

[26] Renard J.B., Dulac F., Berthet G., Lurton T., Vignelles D., Jegou F., Tonnelier T., Jeannot M., Coute B., Akiki R. (2016). LOAC: A small aerosol optical counter/sizer for ground-based and balloon measurements of the size distribution and nature of atmospheric particles—Part 1: Principle of measurements and instrument evaluation. Atmos. Meas. Tech..

[27] Renard J.B., Dulac F., Berthet G., Lurton T., Vignelles D., Jegou F., Tonnelier T., Jeannot M., Coute B., Akiki R. (2016). LOAC: A small aerosol optical counter/sizer for ground-based and balloon measurements of the size distribution and nature of atmospheric particles—Part 2: First results from balloon and unmanned aerial vehicle flights. Atmos. Meas. Tech..

[28] Hering S.V., Friedlander S. (1982). Origins of aerosol sulfur size distributions in the Los Angeles basin. Atmos. Environ..

[29] Whitby K., Husar R., Liu B. (1972). The aerosol size distribution of Los Angeles smog. J. Colloid Interface Sci..

[30] Pope R.M., Fry E.S. (1997). Absorption spectrum (380–700 nm) of pure water. 2. Integrating cavity measurements. Appl. Opt..

[31] Gu F., Yang J., Wang C., Bian B., He A. (2010). Mass concentration calculation with the pulse height distribution of aerosols and system calibration. Optik.

[32] Wang X., Chancellor G., Evenstad J., Farnsworth J.E., Hase A., Olson G.M., Sreenath A., Agarwal J.K. (2009). A Novel Optical Instrument for Estimating Size Segregated Aerosol Mass Concentration in Real Time. Aerosol Sci. Technol..

[33] Giorio C., Tapparo A., Scapellato M.L., Carrieri M., Apostoli P., Bartolucci G.B. (2013). Field comparison of a personal cascade impactor sampler, an optical particle counter and CEN-EU standard methods for PM10, PM2.5 and PM1 measurement in urban environment. J. Aerosol Sci..

[34] Kim J.Y., Magari S.R., Herrick R.F., Smith T.J., Christiani D.C. (2004). Comparison of fine particle measurements from a direct-reading instrument and a gravimetric sampling method. J. Occup. Environ. Hyg..

